# Pharmacist Involvement in Addressing Public Health Priorities and Community Needs: The Allegheny County Racial and Ethnic Approaches to Community Health (REACH) Project

**DOI:** 10.5888/pcd18.200490

**Published:** 2021-01-28

**Authors:** Jennifer Padden Elliott, Stephanie N. Christian, Katie Doong, Hannah E. Hardy, Dara D. Mendez, Tiffany L. Gary-Webb

**Affiliations:** 1 Duquesne University, Center for Integrative Health, School of Pharmacy, Pittsburgh, Pennsylvania; 2 University of Pittsburgh Graduate School of Public Health, Department of Behavioral and Community Health Sciences, Pittsburgh, Pennsylvania; 3 Giant Eagle Pharmacy, Pittsburgh, Pennsylvania; 4 Allegheny County Health Department, Chronic Disease and Injury Prevention Program, Pittsburgh, Pennsylvania; 5 University of Pittsburgh Graduate School of Public Health, Department of Epidemiology, Pittsburgh, Pennsylvania

## Abstract

Community–clinical linkages are connections between community and clinical sectors to improve population health, and community-based pharmacists are well positioned to implement this strategy. We implemented a novel approach to community–clinical linkages in African American communities in which community-based pharmacists implement screenings for chronic disease and social determinants of health, make referrals to clinical and social services, and follow up with patients to support linkage to care in nontraditional health care settings. The community-based pharmacist navigation program works with multisector partners to increase referrals and access to existing health and social service programs. We used a mixed-methods evaluation approach to collect and analyze data on program characteristics and the linkage intervention. From February 2019 to March 2020, 702 African American community members received preventive health screenings, and 508 (72%) were referred to clinical and social services. Pharmacists demonstrated the ability to implement clinical preventive services in nontraditional health care settings and improve access to care through the provision of community–clinical linkages.

**Summary**

**What is already known on this topic?**

The Centers for Disease Control and Prevention published a framework for community pharmacists and physicians to promote community–clinical linkages; most successful models function within traditional health care settings.

**What is added by this report?**

Given multiple barriers to accessing health care in African American and underresourced urban communities, a community-based pharmacist navigation program delivers clinical preventive services including screening, behavioral counseling, and referral to clinical, social, or behavior-change programs, in nontraditional health care settings.

**What are the implications for public health practice?**

With the inclusion of pharmacists in existing payment models, this community-based pharmacist navigation program model can be readily adapted and implemented by other community-based pharmacists and have a major public health impact.

## Introduction

Despite national progress and a narrowing of health gaps by race and ethnicity for some health outcomes, substantial racial and ethnic disparities in health persist for chronic diseases such as diabetes and heart disease (1). In the United States, heart disease is the leading cause of death among African American people, who are 20% more likely than White people to die of a myocardial infarction (2). These trends are similar in Allegheny County, Pennsylvania, the 34th largest county in the United States, with more than 1.2 million people residing in 130 municipalities, including Pittsburgh (3–5). African American people comprise approximately 13% of the population of Allegheny County, yet many communities, especially several in Pittsburgh and others along the rivers, are racially segregated, producing census tracts with a predominately African American population and high poverty rates. Chronic disease rates in the county do not differ significantly from those of the state or nation; however, in census tracts comprising a predominately African American population, rates of chronic disease prevalence are high, driven by health inequities (3).

In 2018, the Allegheny County Health Department received a cooperative agreement from the Centers for Disease Control and Prevention (CDC) as part of its Racial and Ethnic Approaches to Community Health Program (REACH) to implement the Live Well Allegheny: Lifting Wellness for African Americans (LWA^2^) project and work in 6 priority communities in the county to reduce racial health disparities. The LWA^2^ project was developed in response to the most recent community health assessment and a review of community-level data; its leadership includes a diverse coalition of more than 25 partners working together to improve nutrition, breastfeeding support, physical activity, and community–clinical linkages (CCLs) for African Americans living in selected communities (www.livewellallegheny.com/reach). LWA^2^ partners work across sectors and have strong ties with their communities. Although individual behavior changes can improve personal and community health outcomes, system changes are also needed to achieve equity and ensure that residents live well, no matter their zip code or race. Therefore, the coalition advocates for policies that increase access to health care, hospitals, grocery stores, farmers markets, and transportation. The 5-year initiative (2018–2023) bridges the gap between government, schools, churches, nonprofit organizations, and community members of all ages. The goal of the project is the creation of a city where African Americans achieve optimal health and live well.

CDC supports the development of CCLs, which are connections between the community and health care clinics and among other settings where primary care is provided to improve population health (6). Community-based pharmacists practice in settings where patient care is delivered outside the inpatient health system, and they have demonstrated the ability to implement patient care services such as comprehensive medication management, point-of-care testing, and immunization delivery to address various public health concerns (7–9). The Association of American Medical Colleges projects that the demand for primary care providers will continue to exceed the supply (10). As demand for primary care services continues to increase, community-based pharmacists are in a unique position to help fill this health care gap by providing accessible preventive care and CCLs. CDC published a framework for community pharmacists and physicians to promote CCLs; most successful models function within traditional health care settings (11). The physical location of community pharmacies helps improve access to care. More than 90% of the US population lives within 5 miles of a community pharmacy; however, barriers to access to traditional health care settings still exist (12). Given multiple barriers to accessing health care in African American and underresourced urban communities, the Live Well Allegheny REACH Coalition implemented a community-based pharmacist navigation program to deliver clinical preventive services, including screening, behavioral counseling, and referral to clinical, social, or behavior-change programs, in nontraditional health care settings.

## Purpose and Objective

The objective of our study was to describe an evaluation of our novel approach to CCLs. We used a community-based pharmacist navigation program to screen community members for chronic disease and social determinants of health, make referrals to clinical and social services, and follow-up to support the linkage to care in nontraditional health care settings (Figure). Evaluation methods are aligned with the CCL framework from the Agency for Healthcare Research and Quality and focus on program characteristics and the linkage intervention (13). We evaluated short-term outcomes, including number and types of screenings, referrals, and uptake, for February 2019 through March 2020.

**Figure:**
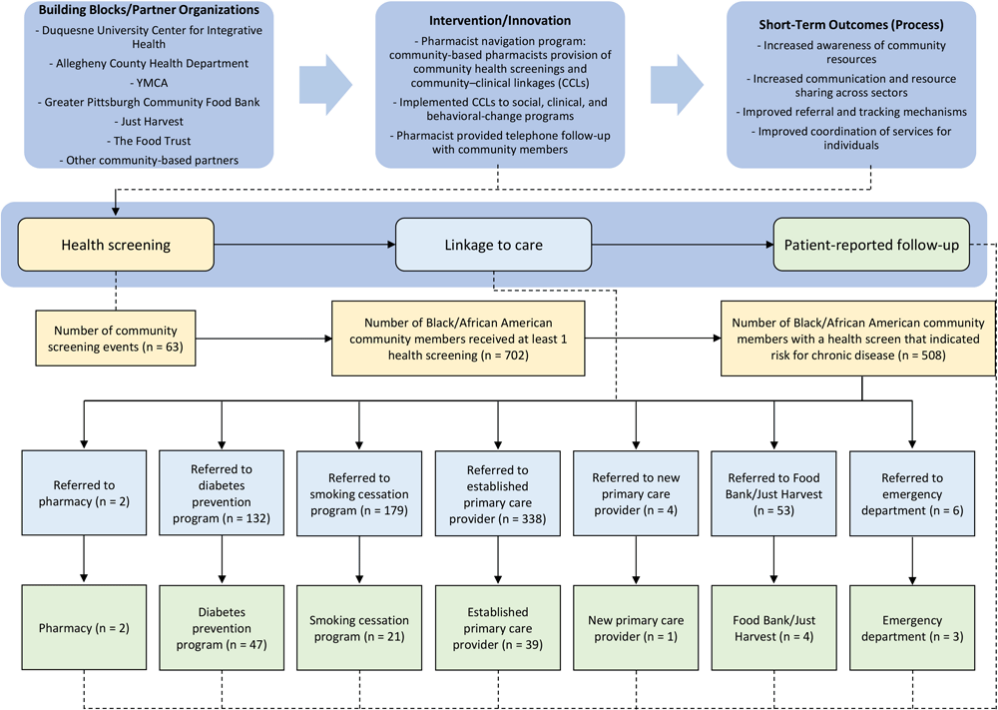
Description of the project, referrals to community resources, and follow-up in a community-based pharmacist navigation program implemented in predominately African American communities, Allegheny County, Pennsylvania, 2019–2020.

## Intervention Approach

Community-based pharmacists practicing in the Duquesne University Center for Integrative Health’s Community Care Clinic implemented the pharmacist navigation program in 6 REACH priority communities. During the planning phase (October 2018–February 2019) Duquesne’s community-based pharmacists worked with the ACHD, federally qualified health centers, community pharmacies, local providers of behavior-change programs (National Diabetes Prevention Program [NDPP], smoking cessation programs), and local food assistance programs to create maps of neighborhood assets. Maps are made available as hard copies and electronically and are updated monthly with current information about programs in each neighborhood. The maps were designed to be easy to read and to accompany all referrals to clinical or social services.

A community-based screening program was designed to include pop-up point-of-care testing for hypertension, diabetes, high cholesterol, and cigarette smoking — all major risk factors for heart disease — and food insecurity screening, counseling, and referral to community services. The Duquesne Community Care Clinic has a Clinical Laboratory Improvement Amendments (CLIA) Certificate of Waiver that allows their community-based pharmacists to perform CLIA-waived point-of-care tests. All health screenings are free to community members and provided by pharmacists, pharmacy practice residents, and student pharmacists, under the supervision of a licensed pharmacist. Pharmacists, pharmacy practice residents, and student pharmacists undergo standardized training on point-of-care testing before providing preventive health screenings at community-based screening events. Pharmacists work with multisector partners to plan health screenings at large community events (festivals, back-to-school events, food drives) as well as regularly scheduled screenings in high-traffic locations in each neighborhood (grocery stores, farmers markets, senior living centers, food distribution sites, YMCAs). Regularly scheduled screenings take place at the same time and location each month to increase access to initial and follow-up health screenings.

Residents aged 18 years or older are eligible to participate in the program. Participants whose screening test indicates chronic disease, chronic disease risk, or food insecurity receive counseling and education from a pharmacist and are referred to one or more of the following: an emergency department for critical values, an established primary care provider, a new primary care provider (if appropriate), a behavior-change program (NDPP, smoking cessation program), a community pharmacy that offers enhanced services, or a food assistance program to help with immediate (food pantries) or long-term needs (enrollment in the Supplemental Nutrition Assistance Program). After the screening event, a pharmacist or pharmacy-practice resident follows up twice via telephone with each participant whose screening test indicated at least 1 chronic disease, chronic disease risk, or food insecurity. The first follow-up call is scheduled 1 or 2 weeks after the initial referral to provide additional counseling, identify any barriers to the referral, and to support linkage to care. The second follow-up call is scheduled 1 or 2 months after the initial referral to evaluate linkage to the referral agency and any additional barriers that can be communicated to multisector partners.

## Evaluation Methods

To evaluate the progress of the activities executed by the community-based pharmacist navigation program and other partners in the REACH coalition, the evaluation team used a mixed-methods approach to collect and analyze data. Monthly coalition meetings, annual data collection on performance measures, key informant in-depth interviews, and focus groups are ongoing. In addition to providing the coalition with necessary updates from funders and the ACHD, monthly coalition meetings allow partners to provide the larger group with updates on their progress and open the discussion for relevant collaborations. Each year, partners provide the evaluation team with performance measures and metrics. For the community-based pharmacist navigation program, Duquesne University Center for Integrative Health reports de-identified data extracted from the Community Care Clinic’s electronic health record system for screening events that took place in priority neighborhoods from February 2019 through March 2020. These data included the number of screening events and locations; where people were referred; the number of people screened; the demographic characteristics of people screened; the types of screening performed; the number of screenings that indicated at least 1 chronic disease, 1 chronic disease risk factor, or food insecurity; and the number of people that self-reported enrollment in applicable programs as a result of the referrals. Screening data were collected at each event and enrollment data were collected during the follow-up process.

REACH coalition leadership had conducted semistructured interviews in person with partner organizations from January 2020 through May 2020 to gather information about the development and progress of coalition activities and availability of community-based resources. The results of the interviews provide insight to better inform and improve coalition strategies. The interviews included questions about each partner organization and its relationship to REACH, its process for planning and development, implementation processes, community relationships, relationships with the larger coalition, challenges and successes, and recommendations for continuing activities. The interviews were audio recorded, transcribed, and later coded by 2 researchers using NVivo qualitative software version 12 (QSR International). After submission to the evaluation team, all data are reviewed, analyzed, and submitted to CDC.

## Results

Preventive health screenings and linkages to care were provided at 63 events in Allegheny County from February 2019 through March 2020. A total of 702 community members who identified as Black or African American received at least 1 health screening, and 508 community members were referred to 1 or more clinical or social services (Figure).

The qualitative analysis of the semistructured interviews revealed 4 dominant themes of the implementation, planning, and development process of the pharmacist navigation program: methods for choosing neighborhood screening events, screening implementation, planning and development of the referral system, and methods for conducting follow-up (Table). During the first year, partners chose to work in neighborhoods that were most familiar to them or those in which they had physical locations. Pharmacists played an essential role in implementing and executing the regularly scheduled health and social determinants of health screenings. The network of REACH partners and community-based organizations constituted accessible referral organizations. Additionally, follow-up calls were conducted methodologically to further support the CCL.

**Table:** Supporting Quotes From Qualitative Interviews on the Implementation, Planning, and Development Process of the Community-Based Pharmacist Navigation and Referral Program

Theme and Findings	Quote
**Methods for choosing neighborhood screening events**	
• Neighborhood choice and frequency of screenings were decided by partners during the first year • Partners decided to work with familiar communities first	There wasn’t always consistency in what health screening was offered, and the locations varied. So part of the planning through REACH [Racial and Ethnic Approaches to Community Health], when we looked at the priority neighborhoods . . . our efforts would be diluted if we tried to start working in all 22 neighborhoods at one time. So we thought, let’s start in the neighborhoods where one of us or a few of us already have relationships and have a presence and develop something together that can then be replicated to other neighborhoods.
	For year one, we chose the Hill District. We had the pharmacy in the Hill for some time, and we still have it. We chose Clairton because a lot of my work has been in Clairton. And we chose Homewood, and that was the Y[MCA] wanted to start in Homewood. And we felt that was good for us because we had 2 sort-of established neighborhoods and a new neighborhood. So that was sort of the process, and it took us probably that first — it wasn’t a full year, but it was kind of like a half a year that first planning year.
	[D]oing regular screenings is also new. So we would do them at different events that people would request. And we had some sites that were regular. But now, we’re being very intentional that we’re there monthly so that people can say, “If you want a free blood glucose test or a free cholesterol, go to here on this day. They’re here every second Tuesday of the month.”
	So our population base really comes from 3 locations. . . . Those are our 3 demographic zip codes that we have chosen to really attack first, mainly because we have branches and sites there that we’re able to work out of. So a lot of our screening opportunities come from there. And we have a good relationship with those communities. . . . We want to make sure that we’re doing right by communities that we’re working in right now. We want to make sure that those efforts are really done to our fullest before we move on. And I think the greatest part about our opportunity here is that we do not have to work inside of our 4 walls. This program is really movable in and throughout the community.
**Screening implementation**	
• Duquesne’s staff played an essential role in conducting screenings and recording data • Screenings were regularly scheduled • Various vitals were taken at the screenings, including the addition of food insecurity screening	So there’s the student pharmacist involvement and the screenings. And then we also do the Hunger Vital Sign screening, which was not part of what we did before, and that happened because of REACH because we’re all brought together and introduced to this.
	We had the intention to do these regular screenings, but it took a while for that to happen, to set up where it was going to be, and whatnot. So it was a lot of maybe the existing relationships that we had and kind of deciding on best partners and times.
	We have really greatly increased the number of health screenings that we’re doing due to wanting to have that presence in our neighborhoods. And because of that, we also had to think of creative ways to be able to do that many health screenings.
	We have monthly screenings. . . . And it is us with the Duquesne pharmacy too, doing blood pressures, CO [carbon monoxide] screenings, and testing glucose screenings. They’re going to do cholesterol every other month. And this is open to the community. Members and nonmembers alike are able to come in.
	Duquesne was willing to step up and say, “Hey, we have the student power. We have 70-plus people that are going to be here. Let us take care of the Excel sheets and bring the data. And, oh, we can do these types of screenings and you can do this. And you put a program here, and we’re going to put one here.” And The Food Trust came and said, “Hey, if there’s individuals that are coming that are hungry, let us give you a food box.” . . . What a great partnership we had.
	I would say that 95% of all screenings that we do are in partnership with them [Duquesne]. So the ability for those data points to be usable across both spectrums is huge because them having a dedicated 10-student team to create these intricate Excel sheets . . . is huge.
	[W]e had worked with Duquesne to actually implement a food insecurity screening question as part of their full assessment that they were doing.
**Planning and development of the referral system**	
• The network of REACH partners and relationships with community-based organizations created opportunities for referrals • Resource accessibility was considered when referrals were made to residents • Referrals were made in real time at the screening events	[W]e created a map of the existing resources in each neighborhood, and we continue to update that as things change and as we learn of new resources.
	[W]e have relationships with the various FQHCs [federally qualified health centers] and providers in those neighborhoods. So we also had been doing the work and knowing that sometimes we were the first person that checked this person’s blood pressure in 10 years and so that we had the ability to identify them and connect them to some of these existing resources. So I think that the maps, we knew, were something that we needed to do first and that we would continually update them as we learned some of these newer neighborhoods.
	[W]e now have the ability for anyone that comes to see us at these screenings to reference any of our partners. If they are there for blood pressure, glucose, CO screenings, if they have questions about their medication, pharmacy, mental health, not seeing a doctor, they can refer them onto a doctor. Food, insecurities, any of those needs, we can reference and get them down. If they have problems at home, not being able to pay their bill, the furnace is down, or air conditioner is down, we can refer them . . . to see if they can get help there. So kind of moving from the first screening we’ve ever had to a place where we’re there to do blood pressure to, now, being able to serve the community.
	We are now spending more time on the marketing, on the focus, on the screenings in these areas where we were, but in the back of our minds, we also knew that this was not an affordable program for someone. And we’re essentially going to do screenings and tell them about a program that they weren't going to be able to take because the Y could not give it out for free. We did not have grant dollars. But now that we know that we can provide this service, it’s a much bigger focus.
	[S]o then we decided like, “Hey, we know Duquesne is going to be here. How about we email them and see if I can partner with them. People go through their screening, after them, you’ll come talk to me, and I'll give you the Food Bucks.”
	[O]ne of the ways that we’ve gotten referrals, particularly for SNAP [Supplemental Nutrition Assistance Program], is for individuals who are screening positive at those health screenings and then need connection to the SNAP team.
	So we’re currently working on a direct referral to . . . a navigator for our programs. So they would work with that individual to connect them to any and all of our programs if they needed it besides SNAP. So that was not something that we went into REACH expecting but it’s been a really great partnership. And Duquesne is doing the screenings at Produce to People. And we’ve just also started some conversation about having them do screenings at a few of what we call our Healthy Pantries. So that’s been exciting and not something that was intended but has allowed us to expand our reach through some of the work that we’re doing with health care providers with Duquesne Pharmacy.
**Methods for conducting follow-ups with referrals**	
• Follow-ups with residents were done in a methodological manner • Follow-ups were used to ensure that residents received care by working with them to guarantee access to services	I think this work gave us the capacity to come together with other partnering organizations to say, “We can do this, and we’ve been doing this. But how can we take it to the next level?” And knowing that these are high-risk patients that might have many reasons why they’re not going to be able to enroll in such and such a program, how can we come together and make it easier? So I think that was the thought behind these follow-ups being very intentional of helping.
	[W]e’ll call 1 time within a week to 2 after the event. And that serves as that kind of extra connection of, “Do you have that referral? What do you think of . . .” and then a month to 2 later for us to say, “Were you able to make it?” and, “Why not?” And only do 2 each time, and if they don’t pick up, we’re just not going to have that data. So that’s where we are, and we are open to anyone’s suggestions. But that was definitely something that we learned and adjusted.
	Another part that is new that I’m really excited about is we will do these screenings, and we would say, “Your blood sugar is 400. You need to go to your doctor.” We will call on critical values. So anyone who had a critical value, we would call them 3 times in the next week until we got a hold of them to make sure that they were connected to care. But for the other patients, we weren’t always doing that consistent follow-up. And so now because of REACH and it being part of it is we’re calling everyone who’s received a health screening and seeing . . . doing the same thing for those critical values, but calling everyone, and calling further out to see, “Did you go to that diabetes prevention program? Did you sign up for SNAP? Did you . . .?” not to make them feel judged if they didn’t, but to say so that we can learn how to better serve the residents, “Why did you go to Hazelwood Health Center?,” but, “Why didn’t you sign up for this?” And then we’re going to try to just keep track of the comments so we can learn what barriers might exist. And we didn’t do that before.

## Implications for Public Health

The community-based pharmacist navigation program implemented in Allegheny County highlights the impact of community-based pharmacists on providing preventive health services and care coordination in nontraditional health care settings in neighborhoods at risk of poor health outcomes. Through the implementation of this model, pharmacists partnered with trusted community-based programs and delivered services in locations frequently visited by residents. This CCL model allows community-based pharmacists to extend their reach outside traditional health care walls to meet community members where they are and decrease barriers to clinical and social services. The effect of the community-based pharmacist navigation program aligns with the effect described in previous studies of pharmacist-provided preventive health screenings and care coordination in traditional and nontraditional health care settings (14,15). The number of community members reached through our program was similar to the numbers described in other work; however, our focus on CCL and the integration of the multisector REACH partners into the referral process was unique. Our model can be easily expanded or modified to include other clinical and social services. Substance use disorder screenings have been added to our model, along with naloxone distribution and connection to medication-assisted treatment programs. Our model also offers the potential to implement other social determinants of health screenings in addition to food insecurity, as long as systems are in place and partners can provide the resources needed.

We faced challenges in documenting patient-reported follow-up with clinical and social services. Some residents did not answer telephone calls and some telephone numbers were disconnected. Additionally, pharmacists initially made both follow-up telephone calls within 2 weeks of the screening event. We quickly determined that although this time frame supported linkage to care, it did not give residents enough time to connect with the referral, and, thus, the short time frame negatively affected accurate documentation of linkage to care. The second follow-up telephone call was changed to 1 or 2 months after the screening event, which improved documentation of linkage to care. This time frame also allowed pharmacists to collect information on remaining barriers that could be shared with the REACH coalition and other multisector partners. As an additional strategy to improve linkages to care and documentation, we implemented data-sharing agreements with local food assistance programs and are currently working on similar agreements with federally qualified health centers.

The process of creating the community asset maps showed few behavior-change programs available to residents in some REACH communities. Additionally, follow-up telephone calls showed that some programs had inconvenient hours and locations. The REACH coalition has since created several virtual chronic disease management and support programs, such as the NDPP, smoking cessation programs, and comprehensive medication management. The opportunity also exists to support community-based pharmacists to implement NDPP in their practice sites, especially in community pharmacies.

Community-based pharmacists can substantially improve access to care through implementation of CCLs in both traditional and nontraditional health care settings. However, reimbursement opportunities are limited for community-based pharmacists to provide enhanced patient care services, such as point-of-care testing. Payment models will need to be developed to ensure community-based pharmacists can continue to develop, grow, and provide patient care services to improve access to care, help fill the need for primary care services, and connect historically hard-to-reach patient populations to resources in traditional and nontraditional health care settings. With the inclusion of pharmacists in existing payment models, this community-based pharmacist navigation program model can be readily adapted and implemented by other community-based pharmacists and have a major public health impact.
